# Role of chemistry in nature-inspired skin adhesives

**DOI:** 10.1039/d5sc01777g

**Published:** 2025-05-30

**Authors:** Xiao Yang, Xiaonan Liu, Yeung Yeung Chau, Xuezhi Qin, Hong Zhu, Liang Peng, Kannie W. Y. Chan, Zuankai Wang

**Affiliations:** a Department of Mechanical Engineering, The Hong Kong Polytechnic University Hong Kong 999077 China zk.wang@polyu.edu.hk; b Hong Kong Centre for Cerebro-Cardiovascular Health Engineering (COCHE) Hong Kong 999077 China

## Abstract

As an essential component of wearable technology, skin adhesion plays a critical role in a wide range of wearable device applications. To maintain effectiveness and safety in daily use, skin adhesives must exhibit strong wet adhesion and high biocompatibility, particularly for devices that remain in contact with the skin for extended periods under humid and dynamic conditions. A comprehensive understanding of skin adhesion's chemical mechanisms is fundamental to advancing this technology. Nature offers valuable inspiration, as numerous organisms have evolved sophisticated chemical and physical adhesion strategies that enable strong and reversible bonding. This review begins by exploring the historical development of nature-inspired skin adhesives, followed by a detailed examination of their performance in moist environments. Particular emphasis is placed on the covalent and non-covalent interactions between adhesive materials and skin surface functional groups, considering both biocompatibility and wet adhesion properties. Additionally, we discuss strategies to mitigate hydration-related challenges alongside an overview of characterization techniques, including mechanical, chemical, and biological testing methods. The classification of nature-inspired skin adhesives into chemical and physical approaches is presented, highlighting their applications in thermal management, energy harvesting, wound care, and transdermal drug delivery. Finally, we identify current limitations and propose design strategies to guide the development of next-generation skin adhesives, providing a clear trajectory for future research.

## Abstract 2

This review examines nature-inspired skin adhesives that exhibit strong wet adhesion and high biocompatibility for next-generation wearable technologies, focusing on the chemical and physical mechanisms, design, and applications.

## Graphical abstract

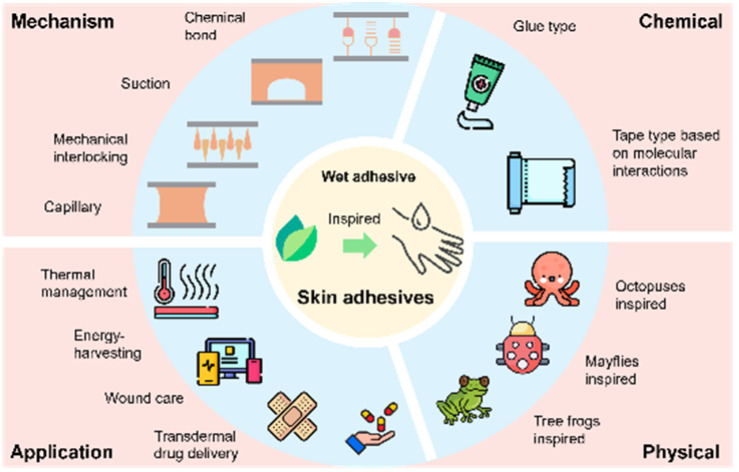

## 1 Introduction

Skin adhesives are integral to the rapidly advancing field of wearable technologies and have become a focal point of research. These adhesives are indispensable for various applications, such as thermal management,^1^ energy harvesting,^2^ wound care,^3^ and transdermal drug delivery.^4^ Skin adhesives must exhibit excellent biocompatibility^5^ and strong wet adhesion^6^ properties to ensure their effectiveness and safety in daily use. Wet adhesion is particularly critical because many applications require prolonged contact between the adhesive and the skin, exposed to moist or dynamically changing environments. Achieving these desired properties necessitates a comprehensive understanding of the mechanisms that govern adhesive performance. This includes chemical interactions as well as physical processes. These insights are crucial for guiding the design of more efficient and reliable skin adhesives, addressing the increasing needs of both medical and consumer technology applications.^7^

Nature provides diverse and efficient adhesion strategies, particularly effective on challenging surfaces such as wet or dynamic skin. Among them, octopuses, mayflies, and tree frogs represent three distinctive biological models that have profoundly inspired the development of synthetic skin adhesives.^8,9^ Octopus suction cups, capable of actively modulating internal pressure, enable reversible and robust negative-pressure adhesion even on wet and smooth substrates, informing the design of suction-based adhesive systems.^10,11^ The soft, mucus-coated tarsi of mayflies achieve stable adhesion to irregular surfaces through a combination of liquid bridging and mechanical interlocking, offering a blueprint for low-modulus, conformable adhesives.^12^ Tree frogs rely on toe pads with hexagonally arranged microstructures and mucus secretion channels to generate capillary forces and viscous fluid bridges, enabling reliable adhesion under wet and rough conditions.^13^ These organisms collectively employ a synergistic integration of chemical bonding, mechanical interlocking, capillary action, and suction mechanisms to overcome the challenges of wet adhesion, dynamic detachment, and substrate adaptability.

By systematically studying these natural systems, researchers have distilled core design principles that guide the development of synthetic skin adhesives. These nature-inspired adhesives have proven especially effective in diverse applications,^14^ where strong, conformal, and durable adhesion on moist or mobile skin is essential. The following sections will discuss how these biological concepts are translated into synthetic systems, emphasizing recent advances in adhesives based on chemical or physical approaches.^15^

**Fig. 1:**
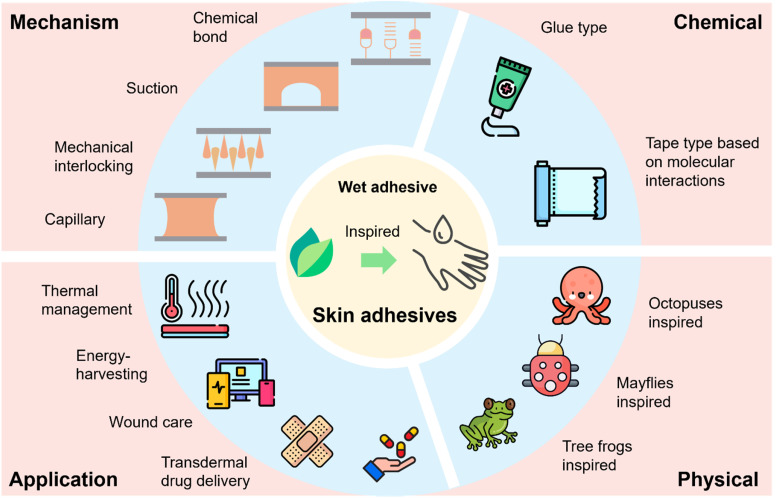
Type, mechanism, and application of nature-inspired skin adhesives.

This review begins by examining the development of nature-inspired skin adhesives and subsequently provides a detailed analysis of the performance of biomimetic adhesives in moist environments. The discussion emphasizes their covalent and non-covalent interactions with skin surface groups, considering both biocompatibility and wet adhesion. Critical strategies for overcoming the challenges associated with hydration are then highlighted to enhance adhesive efficacy. The review continues with an overview of characterization techniques relevant to nature-inspired skin adhesives, encompassing mechanical, chemical, and biological assessments. It further distinguishes between two primary formulation approaches, namely chemical and physical systems, and explores their respective applications in thermal management, energy harvesting, wound care, and transdermal drug delivery. Finally, the review identifies key technological challenges and proposes design strategies for next-generation skin adhesives, offering insights to guide future research efforts (Fig. 1).

## 2 Development of nature-inspired skin adhesives

**Fig. 2:**
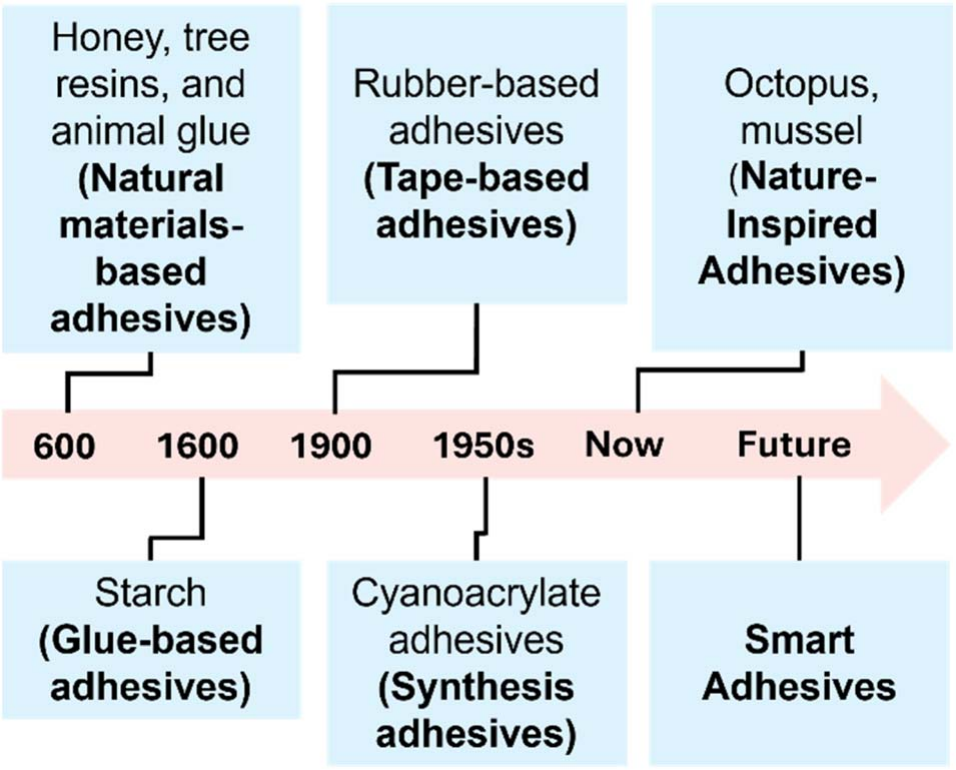
The history of nature-inspired skin adhesive.

As illustrated in Fig. 2, skin adhesives have evolved significantly since at least 600 BCE, when ancient civilizations utilized natural materials such as honey, tree sap, resin, and animal fats for wound closure and infection prevention.^16^ These early wound sealants relied on physical stickiness or simple chemical interactions, such as hydrogen bonding and van der Waals forces, to adhere to skin. The ancient Egyptians, for example, formulated mixtures of honey and animal fat to seal wounds. At the same time, sticky tree sap and plant resins served as early wound dressings by forming semi-permanent adhesive interfaces with skin.

Between the 9th and 16th centuries, European innovations led to various glue-based medical adhesives, including starch-impregnated bandages that stabilized dressings and aided wound healing.^17^ Upon contact with moisture, starch granules swell and gelatinize, forming a sticky hydrogel-like paste that enhances adhesion between the bandage and the skin. In the 19th century, the introduction of rubber-based adhesives further improved flexibility and strength, ultimately leading to the commercialization of adhesive tapes for securing dressings.^18^

A breakthrough came in the 1950s with the development of cyanoacrylate adhesives (often called “superglue”). First discovered in 1942, cyanoacrylates polymerize rapidly upon exposure to trace amounts of water, including ambient moisture or tissue fluids. This anionic polymerization forms strong, durable polymer chains tightly binding to skin and other moist surfaces. Their ability to create robust adhesion within seconds in wet environments earned them the nickname “superglue.” During the Vietnam War, these adhesives were repurposed to control bleeding on the battlefield, demonstrating their potential in emergency wound closure.^19^

In modern times, a new generation of adhesives has drawn inspiration from marine organisms such as mussels and octopuses, demonstrating remarkable adhesion under wet and dynamic conditions. Mussel-inspired adhesives utilize catechol functional groups that form strong covalent and coordination bonds with wet surfaces, representing a chemical adhesion mechanism. In contrast, octopus-inspired designs often feature micro-suction structures that achieve adhesion through reversible physical interactions such as negative pressure and interfacial contact forces.^20^ These nature-inspired strategies highlight the complementary roles of chemical interactions, such as hydrogen bonding, covalent bonding, ionic interactions, and hydrophobic effects, and physical mechanisms in achieving robust and repeatable adhesion on skin.

Recent research also points to hydrogel-based adhesives with high water content and stimuli-responsive behavior (*e.g.*, triggered by temperature,^21^ humidity, or pH^22^), which have shown great promise in diverse applications. Although their high water content typically limits adhesion on wet surfaces, this challenge is addressed by incorporating functional moieties such as catechol groups, aldehydes, or NHS esters that can form covalent or dynamic bonds with tissue surfaces. Additionally, phase-separated or energy-dissipating structures are often employed to enhance interfacial toughness, allowing these hydrogels to adhere firmly and conformably to moist, dynamic biological environments.

## 3 Design of nature-inspired skin adhesives

The development of nature-inspired skin adhesives requires a comprehensive understanding of how adhesion occurs at the skin interface and how it can be maintained under complex physiological conditions. Unlike traditional adhesives, those designed for skin must establish strong yet reversible attachment to a soft, irregular, and often moist surface, while ensuring safety and comfort. To meet these demands, researchers have focused on integrating multiple adhesion mechanisms, tuning material properties for biocompatibility, and overcoming the inherent challenges of wet environments. This section systematically explores the underlying design principles of such adhesives, beginning with the fundamental mechanisms of skin adhesion, followed by key considerations for biocompatibility and skin compatibility, culminating in strategies for achieving effective adhesion under wet or humid conditions.

### 3.1 Mechanisms of nature-inspired skin adhesives

Adhesion at the skin interface arises from synergistic interaction of chemical bonding, suction forces, mechanical interlocking, and capillary effects, all modulated by the skin's surface characteristics and the adhesive.

Chemical bonding involves covalent, ionic, or hydrogen bonds between the adhesive and skin biomolecules, particularly through interactions with the stratum corneum's amino, hydroxyl, or carboxyl groups. These interactions are primarily determined by the surface chemistry of the adhesive, including the type and density of functional groups it presents.^23^

Suction forces generated by negative pressure within confined microcavities at the adhesive and skin interface provide resistance against detachment. These forces are particularly effective on soft, compliant substrates such as skin, where a conformal seal enables the development of pressure differentials.^24^

Mechanical interlocking occurs when adhesives penetrate microscopic topographical features of the skin, such as pores or surface ridges. This interfacial anchoring is promoted by surface roughness, which increases contact area and enhances mechanical retention.^25^

Mediated by liquid bridges between the adhesive and skin, capillary effects promote close contact and interfacial cohesion, especially under humid or moist conditions. These effects depend on the wettability and geometry of the interface, which influence fluid distribution and retention.^26^

Significantly, the elastic modulus of the adhesive critically influences its ability to adapt to the skin's irregular and dynamic surface. Softer adhesives can deform in response to motion and topographical variation, maintaining intimate and continuous contact during movement or deformation.

We have also discussed how these mechanisms are regulated by three key surface characteristics: surface chemistry, surface roughness, and the elastic modulus of the adhesive materials.

### 3.2 Biocompatibility

Biocompatibility is paramount for the daily use of skin adhesives. Safety considerations mandate non-toxic formulations free from substances that can be absorbed through the skin and lead to systemic toxicity.^27^ Common allergens, such as latex and specific resin components, must be avoided to reduce the risk of allergic reactions, and thorough testing is required to confirm that the adhesives do not elicit adverse immune responses like contact dermatitis.^28^ Achieving balanced adhesion without skin irritation often requires maintaining a pH close to the skin's physiological range, approximately 4.5 to 5.5.^29^ Breathability is equally vital for preventing skin maceration; adhesives should allow moisture exchange while remaining intact on the skin.^30^ Biodegradable formulations are preferred whenever possible to reduce environmental impact and the accumulation of nondegradable waste.^31^

Skin adhesives must also accommodate the needs of diverse skin types, including sensitive or compromised skin conditions. Some formulations integrate antimicrobial agents to deter infection risks.^32,33^ The adhesive must sustain its biocompatibility and bonding properties for longer-term applications without degrading the skin. Ease of removal is another critical factor to avoid discomfort or trauma.^34,35^ Finally, regulatory compliance and rigorous clinical evaluations ensure the adhesive's safety and performance across varied populations and environments.

### 3.3 Wet adhesion

Skin adhesives play a central role in ensuring secure and reliable attachment under physiological conditions. However, moisture from bodily fluids introduces substantial obstacles to wet adhesion by preventing the adhesive and the skin from forming intimate contact.^36,37^ Water molecules act as both physical and chemical barriers, segregating the adhesive layer from the skin's surface and impeding key bonding interactions. At the same time, sweat accumulating between the adhesive and the skin can lead to surface contamination or even outcompete reactive groups essential for bonding. Such interference can compromise adhesion strength, reducing immediate bond formation and long-term durability.^38^

Researchers have concentrated on strategies that reinforce chemical interactions between the adhesive and the epidermis to address these issues.^39^ Achieving this goal requires a detailed understanding of skin surface chemistry and a deliberate matching of functional groups in the adhesive to those found on the skin. Covalent bonds, for instance, are formed through reactive functional groups such as *N*-hydroxysuccinimide esters, aldehydes, catechols, isocyanates, or aryl azides, which readily couple with skin amino or hydroxyl moieties. Adhesives that employ these chemistries maintain robust adhesion even in moisture. In addition to covalent crosslinking, non-covalent interactions—such as hydrogen bonding, van der Waals forces, electrostatic attractions, and hydrophobic interactions—contribute to the overall adhesion performance by providing reversible and humidity-tolerant bonding. These mechanisms are particularly relevant in skin adhesives, where pressure-sensitive adhesives often rely on van der Waals interactions combined with the viscoelastic deformation of the adhesive matrix to form effective contact under moist or dynamic skin conditions.

Recent technical advances have focused on mitigating the adverse effects of interfacial water through two primary lines of investigation. The first involves integrating hydrophilic elements, such as hygroscopic polymers, that can absorb or wick away water at the adhesive–skin interface, effectively reducing the thickness of the liquid layer. The second leverages phase-change materials or specialized coatings that modify surface energy in response to environmental stimuli, thereby repelling water or allowing it to disperse. These improvements are particularly beneficial in humid or constantly shifting environments, where maintaining a steady bond poses unique challenges.

Beyond direct chemical and materials engineering solutions, practical design considerations are vital in enhancing wet adhesion. Skin elasticity, for example, varies across different body regions and with user movement, so adhesives must accommodate stretching, bending, and dynamic loads without losing their hold.^40,41^ Individual anatomy further affects adhesion, as structures like folds or hair follicles can change the local surface texture. Adhesive formulations and architectures that account for these real-world variables can better maintain stable, reliable performance over a range of physiological conditions. By merging advanced chemistries with user-centric design, next-generation adhesives have the potential to achieve consistent, high-level adhesion despite the inherent difficulties posed by moisture in daily use.

#### 3.3.1 Chemical regulation of hydration effects

Hydration poses a fundamental chemical challenge in designing skin adhesives, as interfacial water serves as a physical and chemical barrier between the adhesive and the skin surface.^42^ Water molecules separate the adhesive from the tissue and compete with adhesive functional groups for binding sites on the skin, thereby interfering with hydrogen bonding and other molecular interactions. To address this issue, various chemical strategies have been developed to regulate hydration at the adhesive–skin interface, including incorporating hydrophobic domains, water-displacing agents, and moisture-tolerant reactive groups.

An effective approach involves adding hygroscopic or amphiphilic components to the adhesive formulation. These materials can absorb or redistribute interfacial water, thereby thinning the hydration layer and enabling stronger, more direct interactions between adhesive functional groups and skin biomolecules. For instance, hydrogels containing polyethylene glycol segments or zwitterionic polymers can form structured hydration shells while supporting robust interfacial bonding.^43^

In parallel, hydrophobic groups or surface modifiers such as fluorinated moieties, silanes, and alkyl chains can be introduced to repel water and maintain effective contact by modulating surface energy, thereby enabling the adhesive to function under humid conditions.^44^

From an adhesion standpoint, reactive groups capable of forming covalent bonds under moist conditions, such as *N*-hydroxysuccinimide esters, cyanoacrylates, aldehydes, catechols, aromatic azides, and isocyanates, are particularly advantageous. These groups can displace interfacial water and react with amino, thiol, hydroxyl, and carboxyl residues on the stratum corneum to form stable linkages.^45^

Overall, these chemical regulation strategies not only mitigate the detrimental effects of water but also enable strong and durable adhesion under dynamic and high-moisture environments. Integrating such approaches is critical for advancing the performance and reliability of next-generation skin adhesives.

#### 3.3.2 Physical adhesion strategies to overcome hydration barriers

Unlike chemical adhesion, which relies on molecular bonding, often hindered by interfacial water, physical adhesion leverages structural and interfacial design to bypass hydration barriers. A key strategy involves using microstructured surfaces, such as suction cups, nanopillars, or grooves, that actively displace water and establish direct contact with the skin. These structures generate negative pressure or capillary forces that expel interfacial moisture and reduce the thickness of the hydration layer.

Materials with high surface energy or specific wettability can facilitate water displacement and increase real contact area. These mechanisms enable physical adhesives to maintain strong, reversible adhesion even on wet or mobile skin surfaces without relying on covalent or ionic bonding.

Physical adhesives are often implemented in tape-based formats and are particularly advantageous for applications requiring reversibility, gentle removal, and repeated use.

Because physical adhesion does not require molecular-level bonding, it is less affected by hydration, making it well-suited for applications where dynamic interfaces or reusability are prioritized.

### 3.4 Skin functional groups

Designing effective skin adhesives requires pinpointing functional groups such as amino, thiol, hydroxyl, and carboxyl in the skin's stratum corneum.^46,47^ Amino and thiol groups in keratin-containing proteins can form stable covalent bonds with complementary groups in adhesives (*e.g.*, *N*-hydroxysuccinimide esters, aldehydes, catechols).^48,49^ Hydroxyl groups contribute to hydrogen bonding, while carboxyl groups participate in electrostatic and hydrogen-bond interactions.^50,51^ By leveraging these groups appropriately, adhesives can balance high bond strength with compatibility to avoid irritation or damage.^52–54^

#### 3.4.1 Types of covalent bonds formed by skin and adhesive

Skin adhesives establish strong adhesion primarily by forming covalent bonds between functional groups on the skin surface and reactive moieties within the adhesive formulation. Functional skin groups such as amino, thiol, hydroxyl, and carboxyl groups engage with adhesive chemistries, including *N*-hydroxysuccinimide esters, cyanoacrylates, aldehydes, catechols, aromatic azides, and isocyanates, to enhance bonding strength and stability.^55,56^

*N*-Hydroxysuccinimide esters are widely utilized in biochemical applications and adhesive formulations due to their high reactivity with primary amines under physiological to mildly alkaline conditions, forming stable amide bonds. Additionally, these esters can react with thiol groups to produce thioesters, broadening their reactivity and significantly reinforcing adhesive performance. This dual reactivity enables a robust and durable bonding mechanism for skin adhesion.

Aldehyde-functionalized adhesives promote strong and persistent adhesion by forming imine bonds with skin amines through Schiff base reactions. Simultaneously, aldehydes can interact with thiol groups to form hemithioacetals, introducing a reversible bonding mechanism that enhances adaptability in dynamic environments. This structural flexibility not only improves bonding durability but also expands the applicability of adhesives across varying environmental conditions.

**Fig. 3:**
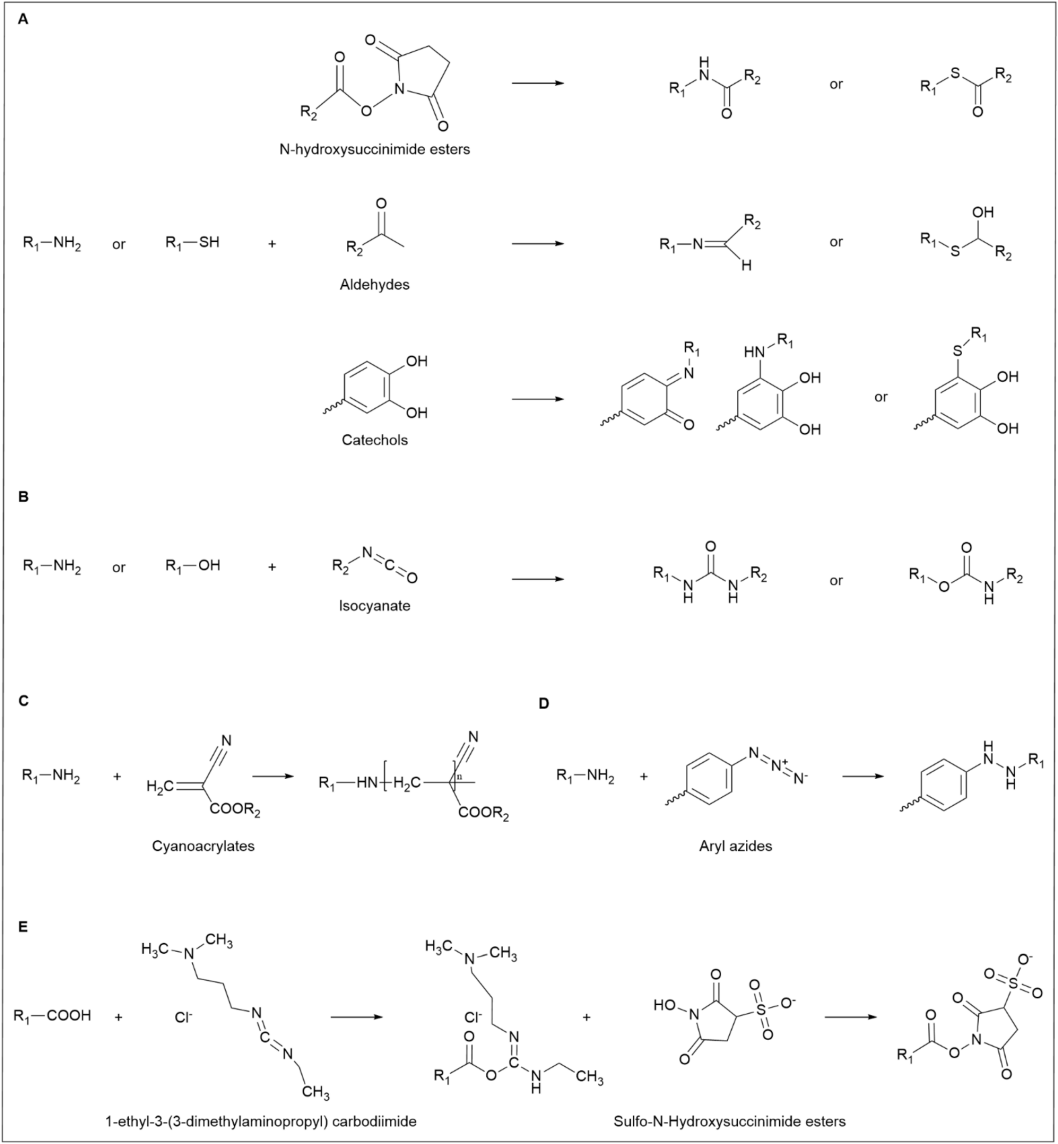
(A) Chemical reactions of amino and thiols with various adhesive groups: *N*-hydroxysuccinimide esters, aldehydes, and catechols. (B) Chemical reactions of amino and hydroxyl with isocyanates. (C) Chemical reactions of amino with cyanoacrylates. (D) The chemical reaction of amino with aryl azides. (E) Carbodiimide cross-linking chemistry.

Oxidized catechol derivatives, such as quinones, contribute to strong adhesion by forming covalent bonds with amine groups *via* Michael addition or Schiff base reactions. Additionally, catechol-based adhesives interact with skin proteins, including histamine, further strengthening adhesion. The thiol–catechol interaction, which involves Michael addition to aromatic rings, enhances bond stability, mimicking the exceptional wet adhesion observed in mussel-inspired adhesion mechanisms. This bioinspired approach significantly improves adhesive functionality in moist environments, offering enhanced performance for medical and everyday applications (Fig. 3A).

Isocyanates react with amine hydrogen to form stable urea bonds, ensuring strong and persistent adhesion under diverse conditions. Simultaneously, they engage in nucleophilic addition with hydroxyl groups, generating urethane bonds that enhance durability and flexibility. These properties make isocyanate-based adhesives particularly well-suited for long-term wear, as they maintain adhesion while accommodating skin movement (Fig. 3B).

Cyanoacrylates interact with amino groups through Michael addition, forming zwitterionic intermediates that polymerize other monomers. This polymerization process establishes robust covalent bonds, significantly reinforcing adhesive strength and ensuring stability under physiological conditions (Fig. 3C).

Aromatic azides generate highly reactive nitrene intermediates upon UV activation, which readily react with skin amines to form stable azo bonds. This photochemically induced bonding mechanism enables durable chemical adhesion, offering a versatile strategy for enhancing adhesive longevity and performance (Fig. 3D).

Carbodiimide chemistry facilitates the cross-linking of carboxyl groups with amino or thiol groups, improving adhesive strength and structural stability. Typically, this reaction employs 1-ethyl-3-(3-dimethylaminopropyl) carbodiimide in conjunction with *N*-hydroxysuccinimide esters to activate carboxylic acids, increasing their reactivity toward amines or thiols. This cross-linking strategy is widely utilized to reinforce adhesive performance by stabilizing functional group interactions and ensuring long-lasting adhesion (Fig. 3E).

#### 3.4.2 Types of non-covalent bonds formed by skin and adhesives

Skin adhesives achieve strong adhesion through a combination of non-covalent interactions, which are crucial in maintaining bonding strength, particularly in moist environments where water can interfere with adhesion.^57^ While each non-covalent force is weaker than covalent bonds, their collective effect significantly enhances overall adhesive performance.

Hydrogen bonding occurs between functional groups such as amines, thiols, hydroxyls, and carboxyls, facilitating rapid adhesion between the skin and adhesive. Although hydrogen bonds are relatively weak and prone to dissociation in wet conditions, such as those involving sweat, their cumulative effect across multiple binding sites provides substantial adhesive strength. To improve stability, hydrogen bonding is often combined with additional adhesion mechanisms. However, adhesives that rely heavily on hydrogen bonds may swell due to their hydrophilic nature, impacting their long-term performance.^58^

van der Waals forces, arising from transient fluctuations in electron density between molecules, are individually weak but contribute significantly to adhesion when acting over large surface areas. These forces maintain higher stability in moist conditions than hydrogen bonds, making them particularly effective for adhesion in humid environments.^59^

Electrostatic interactions, driven by the attraction between oppositely charged molecules or functional groups, generate strong adhesion in dry or low-ion conditions. However, their effectiveness diminishes in aqueous environments due to ion interference. Despite this limitation, electrostatic forces can enhance overall adhesion when integrated with hydrogen bonding or covalent interactions.^60^

Hydrophobic interactions occur when non-polar molecules or regions aggregate to reduce exposure to water. These interactions are relatively strong, particularly in biological adhesives used in environments with sweat. While hydrophobic forces help maintain adhesion in wet conditions, they are most effective when combined with other bonding mechanisms such as hydrogen bonds or van der Waals forces.^61^ By integrating these non-covalent interactions with covalent bonding strategies, skin adhesives can achieve enhanced adhesion even in challenging, moist conditions, addressing critical requirements for both clinical and everyday applications.

#### 3.4.3 Removal of interfacial hydrate layer

When skin adhesives are applied to moist surfaces, water at the interface can substantially hinder bonding by preventing direct contact between the adhesive and the skin. One common approach to tackling this challenge involves using hydrogels, particularly those based on polyethylene glycol or polyacrylic acid, with a high affinity for water. These hydrogels help remove excess liquid that would otherwise diminish or disrupt adhesive interactions by absorbing interfacial moisture. They facilitate stronger adhesion through multiple mechanisms, including hydrogen bonding, covalent cross-linking such as *N*-hydroxysuccinimide ester and amine reactions, and hydrophilic bridging that enables close contact in humid conditions.^62^ This rapid displacement of interfacial water is vital for ensuring reliable attachment in real-world, moisture-rich scenarios.

Additionally, gentle mechanical pressure—on the order of 1 kPa—can enhance adhesion by improving the degree of contact between the adhesive and the skin.^63^ Applying pressure helps expel trapped fluids from the interface, promoting immediate bonding and reinforcing the hydrogels' moisture-absorbing capabilities. Consequently, hydrogel adhesives can achieve robust, secure attachment even in challenging conditions, such as sweaty skin or damp clinical environments.

However, absorbing interfacial water may lead to swelling in hydrogel-based adhesives, posing a risk to long-term performance.^64^ If the material significantly expands, its structural stability and mechanical properties can degrade, diminishing flexibility and adhesion strength. This concern underscores the need to optimize hydrogel composition and cross-link density carefully. Balancing strong water uptake for effective adhesion with minimized volume changes is crucial to maintaining durability.

Despite these potential drawbacks, hydrogels remain highly attractive for clinical and everyday applications on moist surfaces. Their ability to rapidly remove interfering water can be a game-changer for skin adhesives that must function under continuously humid or variable conditions. Ongoing research is focused on formulating hydrogel adhesives with controlled swelling and enhanced cross-linking strategies, aiming to preserve the benefits of water absorption without sacrificing mechanical integrity.^65^ By refining these approaches, future hydrogel-based adhesives may exhibit immediate bonding strength and consistent, long-term adherence in even the most demanding environments.

## 4 Characteristics of nature-inspired skin adhesives

Mechanical, chemical, and biological tests can evaluate skin adhesive properties. Mechanical tests measure adhesion strength and burst pressure, chemical tests assess degradation and swelling, and biological tests determine compatibility with cells and tissues and potential immune responses. By combining these methods, researchers can compare different adhesives, though caution is needed as performance often varies under various environmental conditions.^66^

### 4.1 Mechanical testing

The mechanical properties of a skin adhesive, including tensile strength, lap shear strength, peel resistance, and flat impact resistance, are crucial for its performance in daily applications. These properties ensure that the adhesive effectively bonds to the skin, remains flexible during movement, resists stretching or tearing, and retains its integrity over long-term use. Additionally, the adhesive must conform to the skin's texture and can be easily removed without causing discomfort or leaving a residue. Achieving the optimal balance among these characteristics ensures the adhesive's effectiveness, comfort, and durability in thermal management, energy harvesting, wound care, and transdermal drug delivery.

**Fig. 4:**
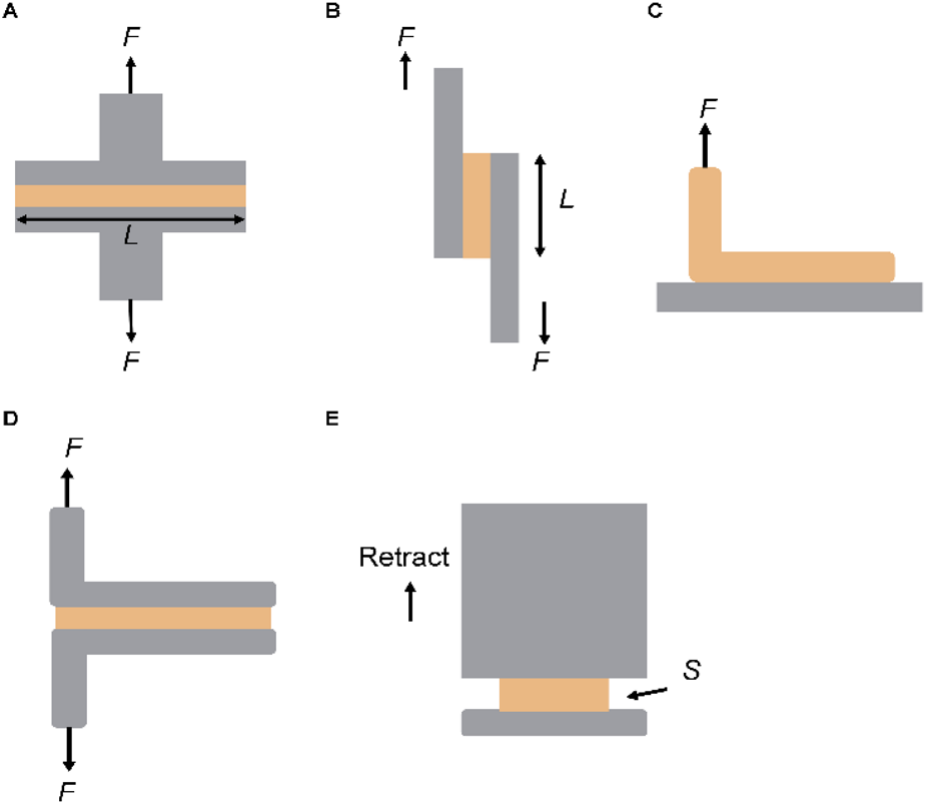
Mechanical testing of skin Adhesives. (A) Tensile test. (B) Lap shear test. (C) 90° peeling test. (D) 180° peeling test. (E) Flat punch test.

The peel test is a widely used method for assessing the adhesive strength of skin adhesives (Fig. 4A). In this test, two substrates are bonded to the adhesive and then pulled vertically to the adhesive interface. The applied tensile force is monitored until the maximum peel force is reached, resulting in separation. In peel tests, the adhesive performance is typically characterized by the steady-state (plateau) peel force, which is normalized by the adhesive width to yield a force per unit width (N m^−1^). This metric reflects the energy required to propagate interfacial failure and is widely used for evaluating soft adhesives on skin or other compliant substrates. While the setup for this test is relatively simple, achieving accurate results requires careful alignment of the samples and precise application of force. This method helps avoid additional stresses, such as bending or twisting, ensuring uniform stress distribution across the sample.^67^ The peel test is also widely used for assessing the toughness of skin adhesives, quantifying the average peel force normalized to the width of the adhesive. This test is particularly suitable for soft adhesives, especially in applications with a risk of adhesive detachment from the substrate. While the preparation process is similar to that of shear tests, focusing on the adhesive's geometry, the primary goal of the peel test is to evaluate the toughness of the skin adhesive by detaching the adhesive from its surface.

In practical applications, skin adhesives are often subjected to shear stress, particularly when the underlying substrates are stretched. To evaluate this, a lap shear adhesion test has been developed, in which two surfaces overlap and are bonded in a defined region (Fig. 4B). Unlike the peel test, this test applies a force parallel to the adhesive interface. Separation occurs when the adhesive interface fails under maximum tensile force. The shear adhesion strength is then calculated by dividing the force by the bonded area. The lap shear test assesses the adhesive's resistance to in-plane stresses by applying a shear force on the bonded substrate. However, adhesive thickness and joint length can significantly influence the results, making geometric considerations essential for reliable evaluation.

During the test, one end of the adhesive is continuously pulled away from the surface at a fixed angle (*y*), typically at a constant speed. The angle *y* is usually chosen as either 90° or 180°, depending on the experimental requirements and the flexibility of the adhesive. Another variation is the T-peel test, in which both adhesive substrates are pulled simultaneously from one end, simulating a separate 90° peel operation. This setup is particularly advantageous when dealing with thin and flexible substrates (Fig. 4C and D).

The force–distance curve obtained from this test typically shows a phase where the force stabilizes, indicating the beginning of the stable peel process (F_peel). In both the 180° and T-peel tests, the energy required for crack propagation (interface toughness) is determined by multiplying the stable force by two and dividing by the adhesive width (measured in J m^−2^ or N m^−1^).

Additionally, it is essential to recognize that the interface adhesion performance of skin adhesives is influenced by both the mechanical and viscoelastic properties of the adhesive and the substrate. This means that the test results are closely related to the separation rate during the test. Skin adhesives are often used with stiffer backing materials to reduce sample stretching during peeling. This is crucial because the peeling process involves the bending of the backing material and the deformation of the adhesive, making the test highly sensitive to the mechanical and viscoelastic properties of the adhesive.

The indentation method is beneficial for studying skin adhesives, especially when analyzing the properties of surfaces with different surface chemistries or materials with heterogeneity. This technique involves applying pressure to a substrate using a probe of a specific shape until a predetermined displacement and/or force is achieved. Depending on the experimental goals, the applied pressure is maintained for a predetermined hold time, ranging from zero to several hours. After the hold period, the probe is withdrawn until completely detached from the substrate.

Researchers can gain insights into various aspects of adhesive performance by analyzing the force-displacement and force–time curves during the loading, holding, and unloading phases. For instance, the loading phase reveals mechanical properties, such as the elastic modulus of the skin adhesive. In contrast, the difference between the loading and unloading phases can indicate the system's hysteresis, where different unloading behaviors may arise from time-dependent physical or chemical interactions. Notably, the peel force (analogous to the maximum adhesion force in tensile and lap shear tests) is a key parameter in evaluating skin adhesive performance.

In indentation tests, researchers often adjust the shape of the probe (*e.g.*, curved or flat, as shown in Fig. 4E). The primary advantage of using a flat punch for planar adhesion testing is that the contact area between the probe and the substrate remains constant and measurable. This is especially useful when evaluating the underwater adhesion performance of skin adhesives, as it eliminates the need for real-time monitoring of the contact area under complex operating conditions. Furthermore, the flat design simplifies the manufacturing process of the test equipment, making it particularly suitable when the adhesive is difficult to shape into a curved surface. Proper alignment between the flat probe and the substrate is crucial to ensure accurate results. It is important to note that high stresses at the edges of the contact area may lead to significant strains, resulting in additional viscoelastic effects.

Multiple mechanical testing strategies have been developed to evaluate the adhesion performance of skin adhesives under various physiological conditions. These methods assess different interfacial properties, including tensile, shear, and peeling strengths, and interfacial toughness under well-defined geometries and loading modes (Table 1). Such comprehensive characterization is essential for correlating adhesive mechanisms with performance outcomes in real-world applications.

**Table 1:** Summary of standard mechanical tests used to evaluate the adhesion performance of skin adhesives

Testing methods	Geometry	Target parameters	Calculation formulas	Features
Tensile test	Width: *W*	Tensile strength (N m^−2^)	*F*_max_/*WL*	Evaluates adhesion strength in the normal direction by pulling apart the bonded surfaces
	Length: *L* (adhesive)			
Lap shear test	Width: *W*	Shear strength (N m^−2^)	*F*_max_/*WL*	Assesses shear direction adhesion strength
	Length: *L* (adhesive)			
90° peeling test	Width: *W* (adhesive)	Interfacial toughness (J m^−2^)	*F*_peel_/*W*	Evaluates adhesion energy and average peel force at a 90° angle and fixed peel speed
180° peeling test	Width: *W* (adhesive)	Interfacial toughness (J m^−2^)	2*F*_peel_/*W*	Evaluates adhesion energy and average peel force at a 180° angle and fixed peel speed
Flat punch test	Contact area between flat probe and adhesive (*S*)	Planar adhesion strength (N m^−2^)	*F*_max_/*S*	Used for load–hold–unload adhesion tests on planar surfaces (contact area remains fixed)

### 4.2 Chemical testing

Chemical testing provides comprehensive insights into skin adhesive composition, structural integrity, and potential degradation pathways. By revealing adhesive components' identity, purity, and stability, these analyses help researchers refine formulations to achieve safer and more effective materials. Among the most commonly employed techniques are nuclear magnetic resonance spectroscopy, Fourier transform infrared spectroscopy, and high-performance liquid chromatography.

Nuclear magnetic resonance spectroscopy is particularly valuable for elucidating skin adhesives' composition and molecular architecture. It identifies various nuclei (^1^H and ^13^C) in the monomer or polymer backbone, clarifying side-chain substitutions, cross-linking density, and overall polymer conformation. In adhesives derived from dopamine-functionalized alginate, for example, NMR can precisely quantify the ratio of dopamine groups to alginate monomers, ensuring that the intended functionalization is achieved. Furthermore, by examining parameters such as chemical shifts and relaxation times, nuclear magnetic resonance offers insight into intermolecular interactions, such as hydrogen bonding, that can profoundly impact adhesive performance. While solution nuclear magnetic resonance is often used for materials dissolvable in specific solvents, solid-state nuclear magnetic resonance techniques enable the investigation of cross-linked or otherwise insoluble adhesives, furnishing a deeper understanding of polymer packing and network organization.

Fourier transform infrared spectroscopy, meanwhile, excels at identifying the functional groups present in skin adhesives and monitoring chemical changes throughout various stages of use or environmental exposure. By measuring infrared radiation absorption at characteristic wave numbers, Fourier transform infrared spectroscopy reveals the presence of essential groups like hydroxyl, carboxyl, or amine functionalities, key to wet adhesion and biocompatibility. This approach also detects newly formed bonds after polymerization or cross-linking and discerns bond scission during degradation. *In situ* Fourier transform infrared setups can observe chemical transitions in real time, assisting in process optimization and quality control. Moreover, an attenuated total reflectance variant of Fourier transform infrared probes the surface of the adhesive, which is especially relevant for studying skin–adhesive interfacial chemistry.

High-performance liquid chromatography offers a powerful means of separating and quantifying individual components in adhesive formulations. By evaluating solvent extracts or dissolved adhesive samples, high-performance liquid chromatography pinpoints residual monomers, cross-linkers, or plasticizers that might affect mechanical properties or biocompatibility. This analysis also facilitates the detection of leached substances or degradation by products over time, an important step in confirming the long-term safety of adhesives in direct contact with the skin. Confirming purity *via* high-performance liquid chromatography ensures the consistency of multi-component formulations, and any detected discrepancies can prompt further refinement before clinical or commercial application.

Beyond these three primary methods, researchers frequently employ complementary techniques such as thermogravimetric analysis and differential scanning calorimetry to assess thermal stability and transition points. X-ray photoelectron spectroscopy can determine surface elemental composition, while mass spectrometry identifies and characterizes low-molecular-weight species in the formulation or its degradation products. These chemical testing strategies deliver a thorough overview of the adhesive's composition, structural evolution, and potential interactions with the biological environment, enabling the design of high-performance skin adhesives that are safe and compliant with regulatory standards.

### 4.3 Biological tests

The biological performance of nature-inspired skin adhesives is typically assessed through a combination of *in vitro* and *in vivo* experiments to evaluate biocompatibility, inflammatory response, and tissue integration. *In vitro* assays commonly involve co-culturing the adhesive materials with fibroblasts, keratinocytes, or stem cells to assess cytotoxicity, often quantified through cell viability (*e.g.*, MTT or live/dead assays)^68^ and proliferation metrics. These assessments follow standardized protocols, such as those outlined in ISO 10993, to support regulatory approval.

*In vivo* studies are essential for capturing the host response in a physiologically relevant environment. Histological staining techniques such as hematoxylin and eosin and Masson's trichrome are used to examine tissue morphology, collagen deposition, and fibrotic responses. In addition, immunohistochemical staining for markers such as CD68 and CD11b enables the identification and quantification of macrophage infiltration, providing insight into immune activation and polarization. For example, a reduced CD68^+^ signal accompanied by increased CD206^+^ (M2 phenotype) expression is indicative of a more regenerative and less inflammatory microenvironment.^69^

Advanced techniques such as flow cytometry and proteomic profiling may be employed to characterize immune cell subpopulations and signaling pathways to gain deeper mechanistic insights. Together, these biological assessments are crucial for ensuring that nature-inspired adhesives are mechanically robust, safe, bioadaptive, and clinically translatable in the dynamic and sensitive environment of human skin.

## 5 Representative examples of nature-inspired skin adhesives

Advances in skin adhesive technologies have increasingly drawn inspiration from natural systems to address challenges such as wet adhesion, mechanical compliance, and biocompatibility. By emulating the key chemical interactions and physical architectures found in biological adhesion, researchers have developed innovative adhesives tailored to human skin's dynamic and irregular surface. This section explores representative examples of nature-inspired adhesives, categorizing them into two primary approaches: chemical and physical. Each approach leverages distinct mechanisms to achieve adhesion, with applications spanning from wound healing to wearable technologies.

### 5.1 Adhesives based on chemical approaches

Chemical approaches to skin adhesion primarily rely on covalent, ionic, or hydrogen bonding to establish strong and durable interactions with biological tissues. These adhesives are designed with reactive functional groups capable of forming stable bonds with skin surfaces. Representative chemical adhesives include glue-type adhesives based on monomers, polymers, coacervates, and hydrogels, and tape-based adhesives based on molecular interactions (such as nanoscale coatings, films, and elastomers). These materials utilize distinct molecular interactions, including covalent cross-linking in polymer networks and electrostatic attraction in coacervates, to achieve robust adhesion under diverse physiological conditions.^70^

#### 5.1.1 Glue-type skin adhesives

Glue-type skin adhesives, including monomers, polymers, coacervates, and hydrogels, are widely used in wearable applications such as thermal management, energy harvesting, wound care, and transdermal drug delivery. These adhesives offer several advantages, including tunable mechanical properties, facile processing, and customizable chemical functionalities.^71^ However, challenges such as biocompatibility, long-term adhesion, and compatibility with dynamic skin surfaces remain unresolved.

Typically, after initial application, these adhesives require a curing period, ranging from minutes to several hours or even days, during which polymerization or cross-linking reactions occur. This curing process forms a stable polymer network, significantly enhancing the adhesive and skin bond. The final material properties, such as elasticity, toughness, and adhesive strength, depend highly on the polymer composition and cross-linking strategy.^72^ This versatility enables glue-type adhesives to be tailored for specific applications, offering reliable performance for a wide range of biomedical and wearable uses.

Monomer-based skin adhesives are polymer monomers or oligomers that polymerize or cross-link after application, forming a robust polymer network. This structure ensures excellent initial adhesion and facilitates uniform distribution on the skin surface, resulting in a strong and flexible bond. Polymerization can be initiated through chemical cross-linking (*e.g.*, UV irradiation) or physical cross-linking (*e.g.*, solvent evaporation or thermal curing). These adhesives perform exceptionally well in wearable skin devices, offering strong and flexible adhesion while maintaining skin comfort. Moreover, they are engineered for long-term stability and reliability, meeting the demands of diverse applications.^73^ Pal *et al.* reported a stable series of poly (α-lipoic acid) adhesives designed for closed-loop recycling, suitable for medical and non-medical applications. By making minor adjustments to the monomer composition, they created pressure-sensitive adhesives that are effective in both dry and wet conditions, with strengths comparable to traditional epoxy adhesives.^74^

Polymer-based skin adhesives can be divided into polymer solution-based and solvent-free polymer melt skin adhesives. Polymer solution-based skin adhesives are created by dissolving preformed high molecular weight polymers in a solvent.^75^ These adhesives capitalize on the inherent properties of the polymers to adhere firmly and flexibly to the skin's surface upon application. Unlike monomer-based adhesives, they eliminate the need for additional polymerization or cross-linking steps, enabling instant adhesion. Adhesion is achieved through physical interactions such as hydrogen bonding, van der Waals forces, electrostatic interactions, and hydrophobic interactions. As the solvent evaporates, the polymer solidifies into a durable, flexible film, ensuring a long-lasting bond.^76^ These adhesives are extensively utilized in wearable skin devices due to their ease of application, instant adhesion, flexibility, comfort, and biocompatibility.

Solvent-free polymer melt skin adhesives have attracted significant attention due to the potential health and environmental risks of organic solvents in traditional formulations.^77^ Achieving good spreadability typically requires low-viscosity polymer melts. However, lower viscosity often reduces cohesion, resulting in weaker adhesion performance.

To overcome this limitation, a widely adopted strategy is to introduce cross-linkable groups into polymers with low glass transition temperatures, thereby enhancing cohesion and adhesion. Furthermore, improving adhesion critically depends on effective surface dehydration, achieved through either of two primary mechanisms: increasing the polymer's hydrophobicity to disrupt the hydration layer or utilizing its hydrophilicity to absorb interfacial moisture.

Coacervate-based skin adhesives leverage the phenomenon of coacervation, where two or more polymers or biopolymers with complementary charges attract each other in solution to form a dense, gel-like coacervate phase. These adhesives are typically composed of natural or synthetic biopolymers, such as gelatin, collagen, chitosan, or alginate, which can form coacervates in the presence of appropriate counterions or other polymers.^78^ Electrostatic interactions between polymers primarily drive the adhesion of coacervate-based adhesives. Due to their gel-like structure, these adhesives demonstrate excellent adhesion in wet or dynamic environments, making them particularly suitable for moist surfaces like skin. Moreover, they offer good biocompatibility, non-toxicity, and high flexibility, ensuring long-lasting adhesion. As a result, coacervate-based skin adhesives are widely used in wearable skin devices and other biomedical applications.

For instance, Narayanan *et al.* developed a mussel foot protein-inspired, tropoelastin-like, bioabsorbable, nonionic self-coacervate polyester by leveraging the low critical solution temperature-driven coacervation phenomenon. This polyester enables the delivery of photo-crosslinked adhesives underwater, overcoming the challenges associated with adhesion in wet or submerged environments. The study demonstrated that these nonionic adhesives can coagulate stably across a broad range of pH and ionic strength conditions and form strong adhesion on underwater substrates in less than 300 seconds. This innovation highlights the potential of smart materials that mimic the self-coagulation properties and environmental stability of mussel foot protein, opening new avenues for the application of bioadhesives in environments with high water content, fluctuating salinity, and varying pH.^79,80^

Hydrogels are widely used as skin adhesives due to their soft, water-rich structure, excellent biocompatibility, flexibility, and strong adhesion on wet or moist surfaces. These materials can be classified into synthetic, protein, and polysaccharide types, forming three-dimensional network structures capable of retaining significant amounts of water. This hydration enhances comfort, reduces skin irritation, and promotes adhesion through molecular mechanisms such as hydrogen bonding, van der Waals forces, electrostatic interactions, and hydrophobic interactions. As a result, hydrogels outperform traditional adhesives, especially on sensitive, fragile, or moist skin surfaces, where they provide better adhesion. Their softness enables them to conform closely to irregular skin contours, ensuring continuous and stable adhesion. This makes hydrogels particularly valuable in applications such as wearable skin devices, where maintaining skin integrity and minimizing discomfort is essential.^81^

However, maintaining adhesion over extended use or under dynamic conditions remains challenging despite these advantages. The high water content in hydrogels can lead to a decline in their mechanical properties over time or cause them to lose adhesion under dehydrating conditions. Researchers have explored various strategies to improve hydrogel formulations to address these issues. For instance, incorporating biomimetic adhesive mechanisms (*e.g.*, catechol) or dual cross-linking approaches has shown promise in enhancing hydrogels' mechanical strength, durability, and adhesion properties. Therefore, hydrogels represent a promising and versatile solution, especially in applications that require reliable yet gentle adhesion.^82^

**Fig. 5:**
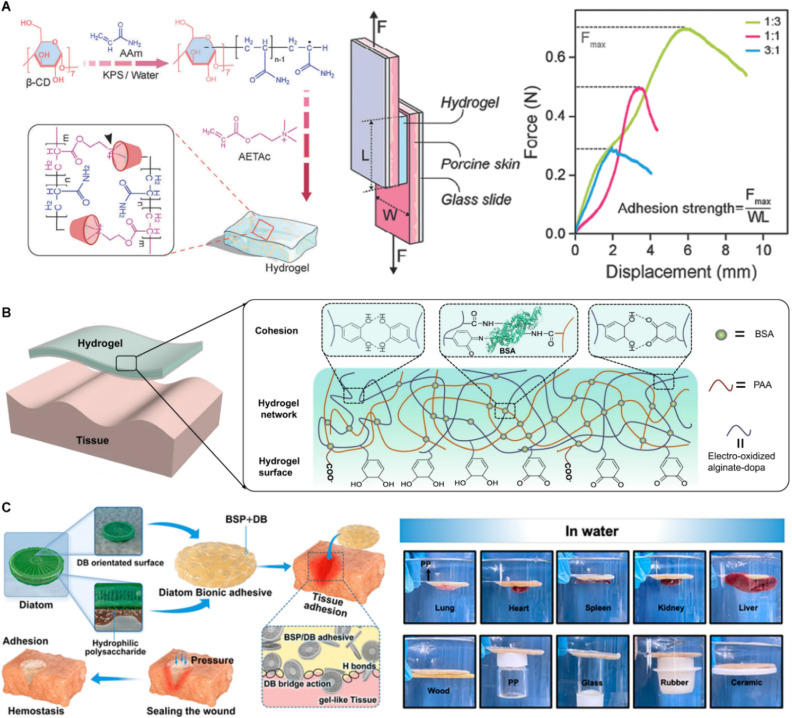
Skin adhesives based on hydrogel. (A) Synthetic hydrogel-based skin adhesives. Reproduced with permission.^83^ Copyright 2024, Wiley. (B) Protein hydrogel-based skin adhesives. Reproduced with permission.^84^ Copyright 2021, AAAS. (C) Polysaccharide hydrogel-based skin adhesives. Reproduced with permission.^85^ Copyright 2023, ACS.

Recent studies have introduced new directions for improving hydrogels. A pseudorotaxane hydrogel composed of polyacrylamide, β-cyclodextrin, and poly(2-(acryloyloxy) ethyltrimethylammonium chloride) bioionic liquid was developed through a supramolecular engineering strategy, resulting in a balanced combination of mechanical toughness (1.1 × 10^6^ J m^−3^), conductivity (≈0.29 S m^−1^), and tissue adhesion (≈27 kPa), along with rapid self-healing and significant stretchability (approximately 3000%) (Fig. 5A).^83^ Additionally, a hydrogel tape was introduced that employs a time-dependent adhesion mechanism, combining immediate, strong wet adhesion with gradual covalent bond formation (Fig. 5B). Inspired by the catechol-based chemical reaction found in mussel foot proteins, electro-oxidation was used to convert catechol to catechol quinone, which then reacted with amines on tissue surfaces, significantly improving the tape's bioadhesion.^84^ Furthermore, a diatom-inspired biomimetic hydrophilic polysaccharide adhesive made from diatom biosilica and Bletilla striata polysaccharide exhibited enhanced bioadhesion and increased cross-linking density in wet environments, demonstrating the substantial potential for hemostasis and wound-sealing applications (Fig. 5C).^85^

#### 5.1.2 Tape-based adhesives based on molecular interactions

Tape-based skin adhesives are soft, solid materials that adhere directly to the skin and perform well in dry conditions. However, their effectiveness in wet environments is often limited due to two main challenges.^86^

First, forming an interfacial hydration layer prevents direct contact between the adhesive and the skin. This water layer disrupts bonding, especially in bulk materials. In contrast, low-viscosity adhesives, such as molecular liquid glues, can better penetrate this layer and form stronger interfacial connections.^87,88^

Second, water droplets can become trapped between the adhesive and the skin, reducing the effective contact area and introducing defects. These issues weaken the overall bonding strength and compromise long-term durability.^89^

To improve wet adhesion, one promising strategy involves the use of adhesives that form molecular interactions with the skin. These adhesives depend on non-covalent forces such as hydrogen bonding, van der Waals interactions, electrostatic attraction, and hydrophobic effects. While generally easier to fabricate and more tunable, they often show lower adhesion strength, particularly in hydrophilic systems.^90^

Common examples of this category include nanoscale coatings, thin films, and elastomers. Their performance is influenced by both material properties and the specific interactions they establish with the skin.^91,92^

Nanoscale coatings are thin, flexible adhesive layers that enhance adhesion through molecular interactions. Materials commonly used include silicone, polyurethane, and acrylic-based polymers, which may also incorporate biomimetic molecules such as catechol to improve wet adhesion. The nanoscale thickness allows the coating to conform evenly to irregular skin surfaces, and its material properties can be tailored to achieve effective interactions with skin proteins, lipids, and moisture. This makes nanoscale coatings particularly advantageous for wearable skin devices. However, challenges remain in maintaining adhesion while avoiding skin irritation, especially when balancing strong adhesion with painless removal. Precise material engineering can provide strong adhesion and comfort, offering a multifunctional solution.^93^

Thin films and elastomers complement each other in molecular interaction-based skin adhesives, enhancing functionality through different material properties and molecular mechanisms.^94^ Thin films, made from polyethylene, polyurethane, or polydimethylsiloxane, are thin, flexible, and provide a smooth and breathable adhesion surface, balancing durability and comfort.^95^ In contrast, elastomers like silicone or styrene-based block copolymers have extremely high stretchability and elasticity, maintaining adhesion even during dynamic movements.^96^ These materials conform tightly to the skin, offering excellent recovery properties and ensuring adhesion under compression.

**Fig. 6:**
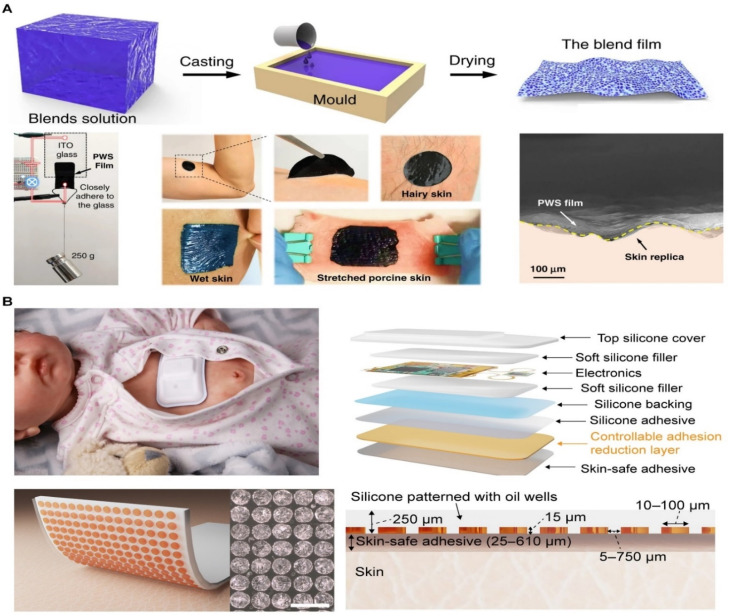
Skin adhesives based on Films and Elastomers. (A) Film-based skin adhesives. Reproduced with permission.^97^ Copyright 2020, Springer Nature. (B) Elastomers-based skin adhesives. Reproduced with permission.^98^ Copyright 2022, AAAS.

Molecular mechanisms like hydrogen bonding, van der Waals forces, electrostatic interactions, and hydrophobic interactions make them well-suited for wearable skin devices. However, challenges persist in optimizing adhesion to wet or irregular skin surfaces, ensuring biocompatibility, and balancing flexibility with ease of removal. For example, Zhang *et al.* developed a highly conductive, organic, self-adhesive, and stretchable dry electrode film made from a biocompatible blend of PEDOT: PSS, waterborne polyurethane, and D-sorbitol (Fig. 6A). This film exhibited excellent conductivity and adhesion on both wet and dry skin.^97^ Additionally, silicone adhesives are commonly used in wound care due to their mild adhesion and biocompatibility, minimizing skin irritation during removal. Jinkins *et al.* proposed a material strategy that wirelessly triggers a reduction in adhesion strength, minimizing skin damage during removal (Fig. 6B). This method involves a silicone composite containing a crystallizable oil, which undergoes a phase change when heated, significantly reducing adhesion at the skin interface.^98^

Table 2 provides a comparative overview based on their chemical adhesion mechanisms, environmental adaptability, mechanical properties, and application-specific advantages and limitations to further clarify the differences between glue-type and tape-type skin adhesives.

**Table 2:** Comparison between glue type and tape-based adhesives based on molecular interactions

Category	Glue type adhesives	Tape based adhesives based on molecular interactions
Representative forms	Monomers, polymers, coacervates, and hydrogels	Nano coatings, films, and elastomers
Adhesion mechanism	Primarily chemical bonding (*e.g.*, covalent, hydrogen, ionic); often enhanced by curing-induced interfacial reactions	Mainly physical interactions (*e.g.*, hydrogen bonding, van der Waals forces); occasional incorporation of chemical moieties for enhanced adhesion
Adhesion formation	Curing or phase transition post-application (*e.g.*, cross-linking)	Instant adhesion without post-curing
Environmental adaptability	Excellent performance in wet/moist or irregular surfaces	Often limited by water layer interference and trapped moisture
Mechanical properties	Tunable *via* polymer composition; high flexibility; potential for self-healing	Generally fixed mechanical strength; less adaptable to dynamic motion
Advantages	Strong and stable adhesion on moist/curved surfaces; customizable properties	Ease of use; conformability; comfortable for dry conditions
Limitations	Requires curing time; may suffer from dehydration over long term	Poor wet adhesion; interfacial water reduces bonding
Applications	Wound dressing, transdermal delivery, bioelectronics, thermal control	Skin-mounted sensors, ECG/EMG patches, motion monitoring

### 5.2 Adhesives based on physical approaches

In contrast to chemical adhesives, physical adhesives typically take the form of tapes and rely on non-covalent interactions such as suction, mechanical interlocking, and capillarity. These strategies, often inspired by biological systems such as octopuses, mayflies, and tree frogs, achieve adhesion through surface structure and physical contact rather than chemical bonding. Key mechanisms include suction, which generates vacuum-based adhesion as seen in octopus' suckers. Mechanical interlocking occurs when adhesives penetrate surface asperities to form interlocked interfaces, as observed in mayfly-inspired surface designs. Capillarity involves liquid adhesives filling micro- or nano-pores and solidifying to form bonds *via* surface tension, mimicking the attachment mechanisms of tree frogs. Owing to their tape-like construction, these physical adhesives are particularly suitable for applications requiring reversibility, gentle removal, and repeated use, especially in dry or mildly moist environments.^99,100^

#### 5.2.1 Suction

Skin adhesives inspired by octopuses utilize a combination of controllable adhesives and embedded sensing, processing, and control systems to manipulate underwater objects precisely. Current synthetic adhesive-based manipulators, in contrast, are typically manually operated, lack sensing and control capabilities, and exhibit slower adhesive activation and release speeds, which limits their manipulation efficiency. To overcome these limitations, an octopus-inspired solution was proposed. Skin adhesives integrated with switchable adhesives, sensing, processing, and control systems enable more efficient underwater manipulation. These adhesives actively adjust adhesive strength through membrane modulation, allowing rapid switching between “on” and “off” states in less than 50 milliseconds—450 times faster than traditional systems. The geometric design of these adhesives allows them to securely adhere to non-ideal surfaces at low pre-tension while independently adjusting their strength and toughness to ensure firm connection and easy release.^101^

**Fig. 7:**
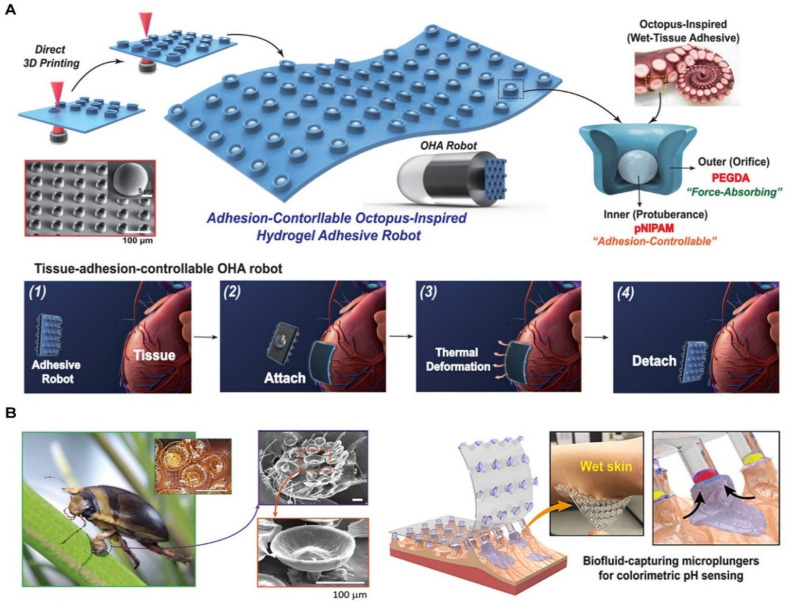
Skin adhesives based on suction. (A) Octopus-inspired skin adhesives. Reproduced with permission.^102^ Copyright 2024, Wiley. (B). Insect-inspired skin adhesives. Reproduced with permission.^108^ Copyright 2021, AAAS.

Additionally, skin adhesives inspired by octopuses have been developed into autonomous, self-healing, multi-layer adhesive patches (Fig. 7A). These patches demonstrate strong adhesion and self-healing properties under dry and underwater conditions. The self-healing structure of octopuses was replicated by developing a dynamic polymer reflow model for 3D patterning of self-healing elastomers. The multi-layer microstructures with varying moduli provide efficient self-healing, reversible adhesion, and stable mechanical deformation. These adhesives adhere strongly to rough skin surfaces and perform effectively in dry and wet conditions. By laminating a thin gold electrode layer onto this octopus-like adhesive structure, skin adhesives can be transformed into self-healing, skin-fitting electronic devices. These devices maintain excellent skin contact, minimize stimulation, and can be reapplied. Such adhesives reliably measure dynamic motion under dry, wet, and damaged conditions, demonstrating their potential for use in wearable technologies.^102–105^

Many practical applications, such as long-term aerial and underwater monitoring, require robots capable of seamless movement between air and water. Li *et al.* described a multifunctional air–water hitchhiking robot that can independently fly, swim, and adhere to surfaces in both environments. The robot's design incorporates a redundant, hydrostatic-enhanced hitchhiking device inspired by the morphology of the remora fish (*Echeneis naucrates*), which allows the device to adhere in a partially attached manner. This device can rapidly cross the air–water boundary in just 0.35 seconds and maintain long-term adhesion with minimal vibration.^106^

Skin adhesives inspired by insect systems enhance adhesion on wet or rough surfaces through oil-bearing spherical cavities and mushroom-shaped tips, optimizing suction effects. Furthermore, research shows that skin adhesives made from highly conformable polymers with multi-scale 3D structures can achieve stable adhesion on soft, wet, and non-flat surfaces, such as skin or organ tissues. Baik *et al.* developed skin adhesives that exhibit enhanced tensile strength and omnidirectional shear resistance, inspired by the hair-like structures of diving beetles (Fig. 7B). By adjusting the diameter of spherical cavities in micro-pillars and forming mushroom-shaped tips, these adhesives exhibit significant improvements in adhesion on both dry and wet skin and organ surfaces.^107,108^

#### 5.2.2 Mechanical interlocking

Inspired by mayfly larvae, skin adhesives based on mechanical interlocking enhance wet adhesion by increasing friction and interlocking between their surfaces and irregular substrates. The larvae utilize various adhesive structures, such as claws on their forelegs, bristle pads on their gills, and spines on their abdomen, to ensure stable attachment underwater. These adaptive features enable them to thrive in both stagnant and turbulent currents. Gorb *et al.* showed that the biological film on the larvae's surface enhances friction with smooth substrates while reducing friction with rough surfaces, indicating the unique role of the biological film in improving adhesion, distinct from the effects of micro/nanostructures.^109^

#### 5.2.3 Capillarity

**Fig. 8:**
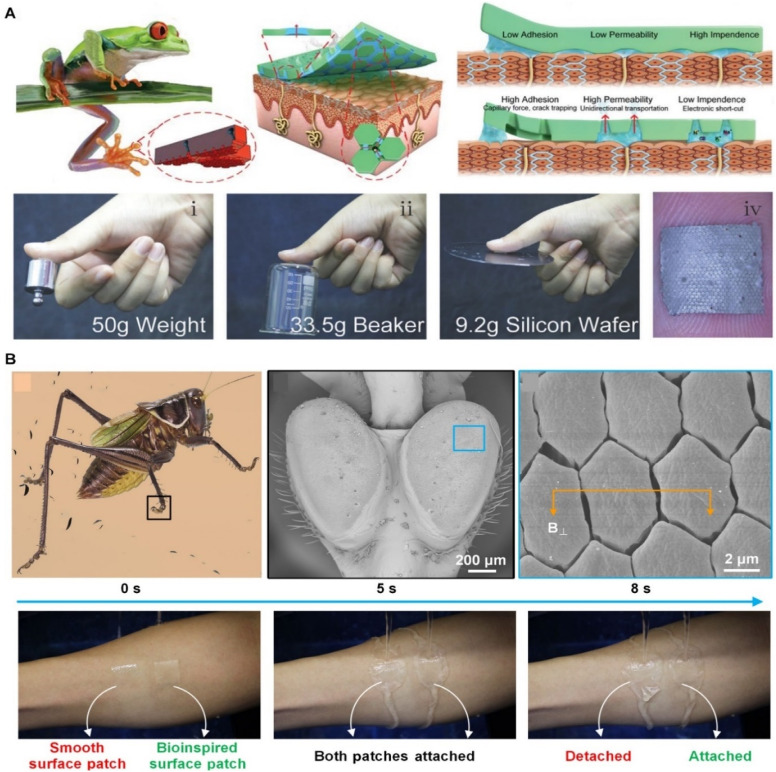
Skin adhesives based on capillarity force. (A) Tree frog-inspired skin adhesives. Reproduced with permission.^110^ Copyright 2024, Wiley-VCH. (B) Chinese cricket-inspired skin adhesives. Reproduced with permission.^111^ Copyright 2023, AAAS.

Skin adhesives inspired by tree frog pads have been designed with bottom-dispersed columns and top-asymmetric conical pores. These structures significantly enhance the contact stability between the adhesive and the surface, improving adhesion 2.79 times in dry conditions and 13.16 times in humid conditions compared to electrodes without such structures (Fig. 8A). Additionally, the enhanced permeable tube design offers significantly higher permeability, 12 times that of cotton. These adhesives demonstrate outstanding durability, being 40 times more durable than commercial Ag/AgCl electrodes. The combination of high adhesion, permeability, and durability makes these tree-frog-inspired skin adhesives highly promising for physiological signal detection.^110^

Furthermore, skin adhesives based on nano-thin liquid bridges, similar to those found in tree frogs, have generated strong wet friction. However, the instability of the nanofluid properties in these bridges has presented challenges in improving wet friction. Researchers discovered that skin adhesives inspired by the Chinese cricket's micro-nano fiber pillars generate stronger wet friction—about 3.8 times higher than tree frogs' columns (Fig. 8B). By introducing a nanofiber pillar array coated with a thin film, skin adhesives enhance friction by reducing interface separation stress and ensuring more stable and larger nano-liquid bridges. These advances have been experimentally validated for use in wearable electronics.^111^

#### 5.2.4 Composite adhesives

Composite skin adhesives are a promising advancement in adhesive technology, combining molecular interactions and biomimetic microstructures. These hybrid adhesives integrate molecular forces, such as hydrogen bonding, van der Waals interactions, and electrostatic forces, with physical microstructural designs inspired by nature. This combination offers enhanced adhesion, durability, and performance, particularly in challenging environments like humid or wet conditions. By leveraging molecular and physical mechanisms, composite adhesives can provide superior functionality, improving adhesion in dry and wet environments. These versatile adhesives are applicable across various fields, including medical wound care and wearable biosensors.^112,113^

For example, a multifunctional skin adhesion interface platform integrates a water-excludable hexagonal array inspired by frog toe pads and an energy-dissipating matrix inspired by snail foot muscle. This design enables strong adhesion in both tensile and shear directions, even under sweating conditions. Microchannels between hexagonal arrays help expel liquid during sweating while enhancing conformal contact with the skin. This system also exhibits excellent anti-vibration performance across a wide range of frequencies (1–150 Hz) in both dry and wet environments, demonstrating its suitability for dynamic conditions.^114^

Another example features a biocompatible adhesive patch with a corrugated, mushroom-like structure, incorporating suction cup-like microcavities that enhance wet adhesion and facilitate biosignal monitoring. Inspired by the microstructure of male diving beetles' forelegs, this design significantly improves multi-directional adhesion on rough skin, both dry and wet, while maintaining high water/air permeability and minimizing skin irritation. A model was proposed to explain how the synergy between suction and capillary action generated by microcavities and micro-wrinkles enhances adhesion, especially in humid environments.^115^

To better understand the design trade-offs among existing skin adhesives, a quantitative comparison is summarized in Table 3. Each adhesive type varies significantly regarding peel strength, shear strength, toughness, and removability, reflecting different design inspirations and clinical applications. These comparisons underscore the need for next-generation adhesives that combine strong yet reversible adhesion, high toughness, and skin compatibility.

**Table 3:** Quantitative comparison of current skin adhesives

Adhesive type	Mechanism/Inspiration	Peel strength (N m^−1^)	Shear strength (kPa)	Toughness (J m^−2^)	Removability	Application	Ref.
Monomer adhesives	Covalent crosslinking upon curing	1000–2000	50–200	Moderate-high	Often irreversible	Rapid wound closure, emergency use	116
Polymer adhesives	Long-chain entanglement, van der Waals forces	300–700	20–80	Moderate	Peelable or dissolvable	Medical tapes, wearable patches	117
Coacervate adhesives	Electrostatic complexation + hydrophobic aggregation	400–1200	40–150	High	Tunable	Wet skin, mucosal adhesion	118
Hydrogel adhesives	Hydrogen bonding, electrostatics, catechol	300–1000	30–100	>500	Partial	Wet-tissue sealing, wearable sensors	119
Nano coatings	Nanoscale surface roughness + van der Waals forces	200–500	15–50	Low-moderate	Easy to detach	Conformal electronics, low-trauma removal	120
Film	Pressure-free adhesion *via* interfacial forces	100–300	10–40	Low-moderate	Easy peel	Bioelectronics, skin-conformal sensors	121
Elastomer adhesives	Viscoelasticity, conformal contact	200–600	20–80	∼100	Designed for easy removal	Long-term skin patches	122
Octopus-inspired adhesive	Microcup suction + capillarity	>1000	>100	Moderate	Pressure-switchable	Underwater or mobile skin attachment	123
Mayfly-inspired adhesive	Soft cuticular microstructure	∼500	∼50	Moderate	Gentle removal	Hairy or fragile skin regions	109
Tree frog-inspired adhesive	Toe pad microchannels, capillarity	500–800	30–60	Moderate	Passive, conformal	Moist, curved, or irregular surfaces	124

## 6 Application of nature-inspired skin adhesives

Due to their versatile properties, nature-inspired skin adhesives have found a wide range of applications beyond traditional medical use. These materials offer innovative solutions in various fields by mimicking natural adhesives that are optimized for biological environments. This section explores the diverse applications of nature-inspired adhesives, focusing on four key areas: thermal management, energy harvesting, wound care, and transdermal drug delivery. These applications highlight the potential of bioinspired adhesives to enhance the functionality and performance of next-generation wearable technologies, medical devices, and other advanced systems.

### 6.1 Thermal management

Skin adhesives play a pivotal enabling role in thermal management for wearable devices by ensuring stable integration of functional materials with the skin.^125–127^ While thermal regulation is primarily achieved through incorporated materials such as aerogels, hydrogels, or silver nanowires, the adhesive serves as a critical interface that provides durable skin attachment, mechanical compliance, and long-term user comfort. These properties are essential for the sustained performance of thermal management systems. Thermal management strategies typically fall into two categories: passive and active approaches.^128,129^

**Fig. 9:**
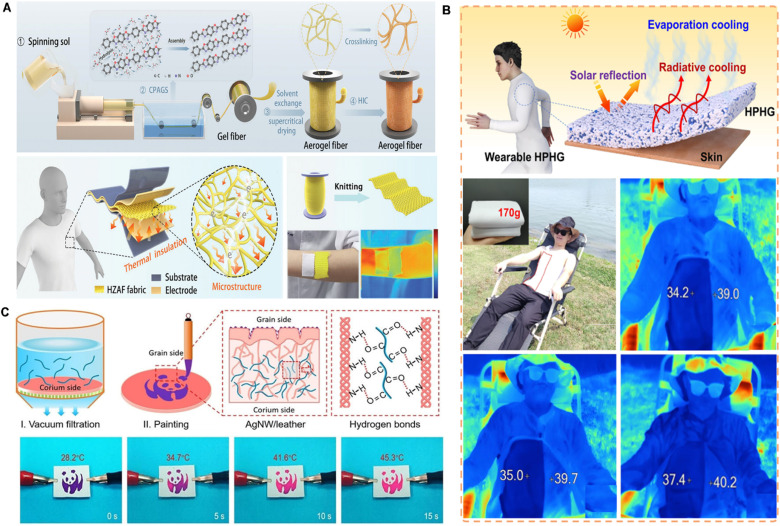
Skin adhesive for wearable thermal management devices. (A) Photos and device structures of polymer aerogel fibers for passive thermal management. Reproduced with permission.^130^ Copyright 2024, Elsevier. (B) Silver nanowire decorated leather with hierarchical structures for integrated visual Joule heating. Reproduced with permission.^131^ Copyright 2022, Wiley-VCH. (C) Schematic illustration of textiles based on zylon aerogel fibers for self-powered sensing in harsh environments. Reproduced with permission.^132^ Copyright 2024, Wiley-VCH.

In passive thermal management, skin adhesives incorporate thermally insulating materials, such as aerogels and layered hydrogels, to reduce heat transfer and enhance user protection in extreme environments. For instance, polymer aerogel fibers composed of crosslinked nanofibers demonstrate outstanding flame resistance (up to 650 °C) and low thermal conductivity, making them ideal for textile-based wearables in harsh settings (Fig. 9A).^130^ Additionally, porous hydrogel structures can maintain surface temperatures below ambient levels, significantly extending cooling duration under thermal stress (Fig. 9B).^131^

In active thermal management, adhesives can host phase-change materials that absorb or release heat in response to temperature changes. For example, flexible composite materials that integrate paraffin with olefin block copolymers and SEBS form a dual 3D cross-linked structure, offering improved thermal regulation, mechanical integrity, and leakage resistance—features essential for wearable thermal therapy.

Moreover, integrating multifunctional materials into the adhesive layer allows for additional capabilities, such as Joule heating, electromagnetic shielding, or piezoresistive sensing. While these functionalities arise from embedded components like silver nanowires or leather composites, the adhesive matrix ensures their stable performance on the skin by conforming to dynamic motions and maintaining intimate contact (Fig. 9C).^132^

Although the skin adhesive is not the primary functional material, its mechanical robustness, biocompatibility, and interface stability are vital for the effective application of thermal management systems in wearable electronics. As fabrication techniques advance, skin adhesives will continue to evolve as an adaptable platform for integrating thermal regulation into next-generation biomedical and AI-driven wearables.^133^

### 6.2 Energy-harvesting

Wearable electronics, such as smartwatches, fitness trackers, and health monitoring systems, have been widespread in medical and daily life. An untethered power supply to functional components guarantees sustainable operation for key elements of these devices.^134,135^ Flexible and stretchable energy storage devices are becoming optimal choices for next-generation wearable technology. Their ability to seamlessly integrate with wearable systems and conform closely to human skin makes them well-suited for enhancing functionality and user comfort.^136–138^

Skin adhesives play a critical role in wearable energy storage devices by maintaining stable contact between the device and the skin, ensuring consistent functionality. A wide range of adhesive materials is used for this purpose, including commercial tapes, biomaterials such as hydrogels, and stretchable polymer materials. The selection of an appropriate adhesive depends on factors such as flexibility, durability, biocompatibility, and Influence on battery output, all of which contribute to optimizing the performance of wearable energy storage systems.

**Fig. 10:**
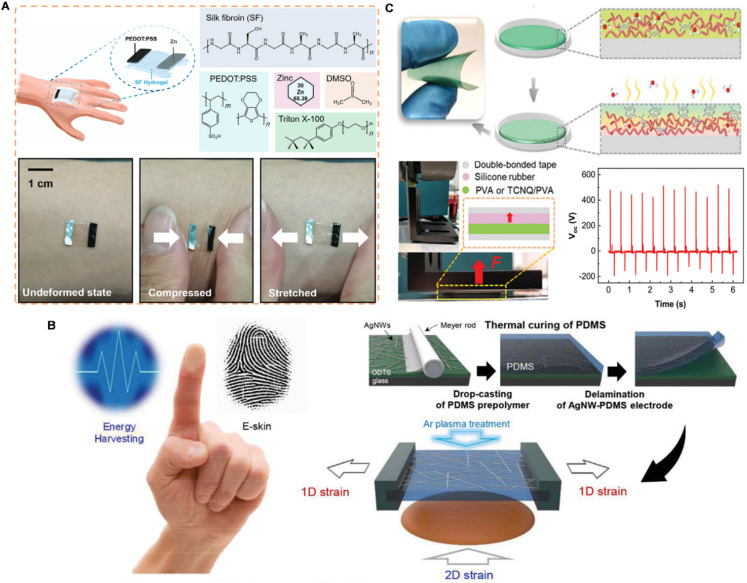
Skin adhesive for wearable power devices. (A) Photos and device structures of silk fibroin hydrogel based wearable stretchable battery. Reproduced with permission.^141^ Copyright 2024, Wiley-VCH. (B) Logic diagram of fingerprint-inspired energy-harvesting electronic skin. Reproduced with permission.^149^ Copyright 2019, Wiley-VCH. (C) Schematic illustration of a TCNQ/PVA blend film based flexible biomechanical energy harvester. Reproduced with permission.^150^ Copyright 2022, Wiley-VCH.

Commercial tapes are widely used in wearable devices due to their softness and strong adhesion to the skin. For instance, medical-grade adhesive films (3M medical tape 1524) have secured microfluidic systems for physiological signal monitoring in sweat-activated biocompatible battery devices.^139^ Stretchable polymers, such as polydimethylsiloxane, are commonly utilized as adhesive layers in wearable electronics. A notable approach involves the fabrication of micropillar arrays on polydimethylsiloxane surfaces, which enhance adhesion by mimicking the gecko-inspired adhesion mechanism. This biomimetic adhesive has been integrated with flexible micro-supercapacitors and strain sensors to form multifunctional wearable systems. When attached to the skin, these devices can detect biological signals, including arterial pulse, swallowing, and facial muscle movements, using the energy stored in the supercapacitor.^140^ Biomass-based materials also serve as effective adhesives in flexible wearable devices due to their degradability, permeability, biocompatibility, and adjustable adhesion properties. Silk fibroin hydrogel, for instance, exhibits both solid-like structural integrity and liquid-like adaptability while maintaining excellent ionic conductivity. A lateral design approach has been employed to directly integrate conductive polymer-based positive and zinc-based negative electrodes into a silk fibroin ion hydrogel membrane. This configuration supports the skin's natural breathability while ensuring stable electrical performance under mechanical stresses such as bending, stretching, and twisting, thereby preserving comfort and functionality during movement (Fig. 10A).^141^

Energy harvesting technology converts human-generated energy into electrical power as a complementary or alternative solution to traditional wearable energy storage systems. This advancement can reduce battery size or even eliminate the need for batteries.^142^ By extending device usage and minimizing the risks associated with battery replacement, such as infection and user inconvenience, this approach enhances both functionality and user experience. Wearable energy-harvesting devices have been extensively developed using various mechanisms, including piezoelectric,^143^ triboelectric,^144^ thermoelectric,^145^ and dielectric elastomer-based systems.^146^ These technologies effectively capture the thermal and mechanical energy produced by the human body, providing a sustainable power source for wearable electronics.

The adhesion layer in these devices plays a dual role by securing the device to the skin while facilitating energy collection and transmission. However, mechanical interference from the adhesive layer can create inconsistencies at the interface between the electronic device and the skin, potentially leading to motion artifacts in wearable bioelectronic devices and increasing the risk of inaccurate output.^147^ To mitigate these issues, the selection of adhesive materials is typically limited to stretchable polymers, often integrated with conductive components. Additionally, optimizing the structural design of the adhesive enhances both adhesion strength and energy-harvesting efficiency.

Integrating the bioanode and biocathode into a hydrogel substrate creates an “island-bridge” structure, where the “island” collects and stores energy from sweat. At the same time, a liquid metal component functions as a “bridge” connecting the system. This configuration ensures a secure fit against the skin while maintaining exceptional adaptability and stability during movement and stretching.^148^ Inspired by fingerprint patterns, polydimethylsiloxane wrinkles are the primary microstructure, with embedded silver nanowires forming secondary nanostructures to create conductive hierarchical wrinkles. This conductive layer adheres directly to the skin, acting as an energy collection interface within triboelectric nanogenerators. The design conforms closely to the skin surface, significantly improving the efficiency of mechanical energy harvesting and enabling precise self-powered pressure sensing (Fig. 10B).^149^ A hybrid film has also been developed by incorporating 7,7,8,8-tetracyano-p-xylidine into a polyvinyl alcohol matrix, allowing for a controlled transition between adhesive and non-adhesive states. During contact, the surface charge increases in proportion to adhesion strength. However, stronger adhesion increases interfacial binding energy, leading to more significant separation stress. This innovative switch balances the benefits of both adhesive and non-adhesive interactions, achieving a record peak power density of 20.5 W m^2^ Hz^−1^ at a low matching impedance of 1 MΩ (Fig. 10C).^150^

### 6.3 Wound care

Skin adhesives are crucial in managing wounds resulting from surgery, chronic diabetes, and burns, as scars can significantly impact appearance and psychosocial well-being. It is estimated that approximately 100 million people worldwide suffer from surgical or traumatic scars, which can range from fine linear scars to more severe forms, such as hypertrophic scars and keloids.^151^ Effective scar management aims to reduce the visibility of scars, minimize the adverse effects of abnormal scarring, and promote rapid healing. Skin adhesives have been demonstrated to help stop bleeding, stimulate tissue regeneration, and reduce allergic reactions. Natural materials like sodium,^152^ alginate,^153^ chitosan,^154^ gelatin,^155^ and hyaluronic acid^156^ are pivotal in wound care due to their biocompatibility and biodegradability.

Wound healing typically progresses through four stages: hemostasis, inflammation, proliferation, and maturation.^157^ The smooth progression of each stage is crucial for optimal wound repair, and skin adhesives play an essential role in supporting these processes by enhancing hemostasis, reducing inflammation, and promoting tissue regeneration and angiogenesis.^158^

During the hemostasis phase, skin adhesives exhibit excellent hemostatic properties. For example, the collagen-starch hydrogel adhesive developed by Yang *et al.* demonstrates strong wound closure capabilities, efficient red blood cell blocking, and activation of hemostatic barrier membranes, outperforming traditional fibrin glue in hemostatic performance.^156^ Similarly, a modified hydrogel composed of chitosan, sodium alginate, and tannic acid, developed by Zou *et al.*, demonstrated significantly improved hemostatic efficiency compared to commercial products, particularly in a rabbit liver injury model. This was due to the enhanced antibacterial and antioxidant properties of the hydrogel.^159^

In the inflammatory stage, skin adhesives are essential in inhibiting excessive inflammation and promoting tissue regeneration. These adhesives achieve this by reducing inflammation and accelerating tissue repair through their antibacterial and antioxidant functions. For instance, mussel-inspired biomimetic hydrogel adhesives have been shown to optimize mechanical properties and adhesion, effectively treating diabetic oral wounds by reversing reactive oxygen species-mediated immune disorders.^160^ Additionally, bioadhesives based on silk fibroin and silver nanoparticles further accelerate wound healing by exhibiting self-healing and antibacterial properties, as well as promoting tissue regeneration.^161^

The proliferation phase, characterized by fibroblast proliferation and angiogenesis, is crucial for wound repair. Skin adhesives support this phase by promoting angiogenesis, facilitating fibroblast migration, and stimulating collagen synthesis. For example, the snail mucus-derived bioadhesive developed by Deng *et al.* exhibited remarkable hemostatic activity, biocompatibility, and biodegradability.^162^ This adhesive accelerated skin wound healing in diabetic rats and promoted macrophage polarization toward an anti-inflammatory phenotype, significantly improving epithelial regeneration and angiogenesis.

In the final maturation stage, skin adhesives are critical for maintaining a moist environment and occlusion to prevent hypertrophic scar formation.^163^ Proper moisture retention and occlusion reduce excessive collagen deposition, minimizing scar hyperplasia.^164^ Mucosa-inspired hydrogels effectively maintain a moist environment and reduce scar formation. Furthermore, biomimetic membrane materials, which mimic the fetal extracellular matrix, help minimize inflammation and promote collagen reconstruction, further mitigating scar formation.^165^

By utilizing biomimetic and nature-inspired adhesives, wound healing can be significantly enhanced, and scar formation can be prevented. These adhesives improve tissue regeneration, provide anti-inflammatory and antibacterial benefits, and maintain a moist microenvironment, which is crucial for effective wound care. Therefore, adhesives derived from natural materials hold great promise for wound care applications.

### 6.4 Transdermal drug delivery

Skin adhesives play a crucial role in transdermal drug delivery (TDD), directly contacting human skin to facilitate the effective entry of drugs into systemic circulation. TDD, a non-invasive method of delivering drugs through the skin, offers several advantages over traditional oral and intravenous administration. It ensures the secure anchoring of drug patches, supports controlled release into the bloodstream, and bypasses the digestive system. This method is non-invasive and conducive to self-administration, also reduces gastrointestinal side effects, thereby improving patient compliance.^166^ Additionally, it eliminates the need for needles, lowering the risk of cross-infection and minimizing medical waste. The system can provide sustained, controlled drug release for up to one week. Due to their simple structure and ease of use, drug-adhesive patches have become widely adopted in TDD systems.^167^

The skin adhesive TDD system significantly improves drug bioavailability, ensuring efficient pharmacokinetics with minimal invasiveness. However, variations in drug properties, such as molecule size, hydrophilicity, lipophilicity, and charge, often require chemical enhancers to increase skin barrier permeability.^168^ These enhancers offer additional driving forces to help drugs penetrate the skin while minimizing damage to deeper tissues. For example, magnesium microparticles have been successfully employed as micromotors in microneedle delivery platforms, facilitating deeper and faster intradermal drug delivery. These micromotors generate hydrogen bubbles rapidly, providing sufficient force to disrupt the dermal barrier and enhance the delivery of therapeutic payloads.^169^

A significant challenge for skin adhesives in TDD is achieving effective penetration of the skin surface barrier while maintaining strong adhesion, particularly in fluid environments. Inspired by marine blue mussel foot proteins, polydopamine has gained significant attention for its excellent adhesive properties. Rich in catechol groups, polydopamine forms covalent bonds with amino or thiol groups on tissue surfaces *via* Michael addition and Schiff base reactions, thereby enhancing adhesive strength.^170^ For instance, embedding mesoporous silica nanoparticles into polyacrylamide/polydopamine hydrogels has significantly improved both mechanical strength and adhesion, facilitating efficient transdermal drug delivery in *in vitro* studies.^171^

Nature-inspired nanostructures further enhance the adhesive performance of chemical TDD patches. For example, nanoporous hydrogel adhesive patches establish tight contact with the skin while releasing maltol. These patches, with asymmetric adhesive structures inspired by the surface properties of diving beetles, demonstrate significantly improved adhesion.^172^ Similarly, cup-shaped structures modeled after octopus suction cups achieve strong wet adhesion in liquid environments. These designs protect internal chemical bonds and leverage air pressure differentials to provide additional physical adhesion.^173^

Nanostructures significantly enhance the adhesive performance of transdermal patches by increasing surface contact, enabling mechanical interlocking, and incorporating functional modifications. These advancements pave the way for developing efficient and robust transdermal drug delivery systems.

## 7 Conclusion

Inspired by natural adhesion mechanisms, chemical innovations drive breakthroughs in biomimetic skin adhesives. Researchers have developed adhesives with strong adhesion, remarkable integration, and excellent environmental adaptability by replicating biological adhesion strategies. These adhesives have been widely applied in thermal management, energy harvesting, wound care, and transdermal drug delivery.

Despite significant progress, biomimetic skin adhesives face challenges, particularly in practical applications in biomedicine and wearable devices. While existing strategies have made substantial advancements in optimizing adhesion, long-term stability, and performance in dynamic, humid environments, there is still room for improvement. Overcoming these key technical bottlenecks, such as improving adhesion strength under wet or dynamic conditions, enhancing material durability, and ensuring scalability for mass production, will drive broader applications in flexible electronics, biomedicine, and sustainable technologies.

Recent advancements in materials science are expanding the potential of biomimetic skin adhesives. Stimuli-responsive materials, phase-change compounds, and self-healing polymers provide dynamic adaptability, while conductive nanomaterials and thermosensitive compounds enhance comfort and responsiveness. These innovations drive their integration into healthcare, smart sensing, flexible electronics, and energy management. Additionally, advancements in manufacturing technologies allow for the precise fabrication of biomimetic structures, providing application-specific solutions that enhance both performance and versatility. The ability to engineer adhesives at the micro and nanoscale further refines their functionality in various domains.

Looking forward, interdisciplinary collaboration will be essential in optimizing biomimetic skin adhesives. Integrating biology, chemistry, materials science, and artificial intelligence will propel the development of next-generation intelligent adhesives, fostering innovations in medical devices, wearable electronics, and environmental applications. A deeper understanding of micro-scale regulatory mechanisms will establish the theoretical foundation for high-performance, sustainable adhesive materials, ultimately expanding their applications and enhancing human quality of life.

## Author contributions

X. Y., X. L., and Y. Y. C. have the same contribution. All authors have approved the final version of the manuscript.

## Conflicts of interest

There are no conflicts to declare.

## Data Availability

No primary research results, software or code have been included and no new data were generated or analysed as part of this review.
